# Supplementary figures and images for: Effects of Lactobacillus plantarum HW1 on Growth Performance, Intestinal Immune Response, Barrier Function, and Cecal Microflora of Broilers with Necrotic Enteritis
Source: Animals (Basel). 2023 Dec 10;13(24):3810. doi: 10.3390/ani13243810 (PMC10740588; doi:10.3390/ani13243810)

Supplementary Figure S1. Western blots raw data

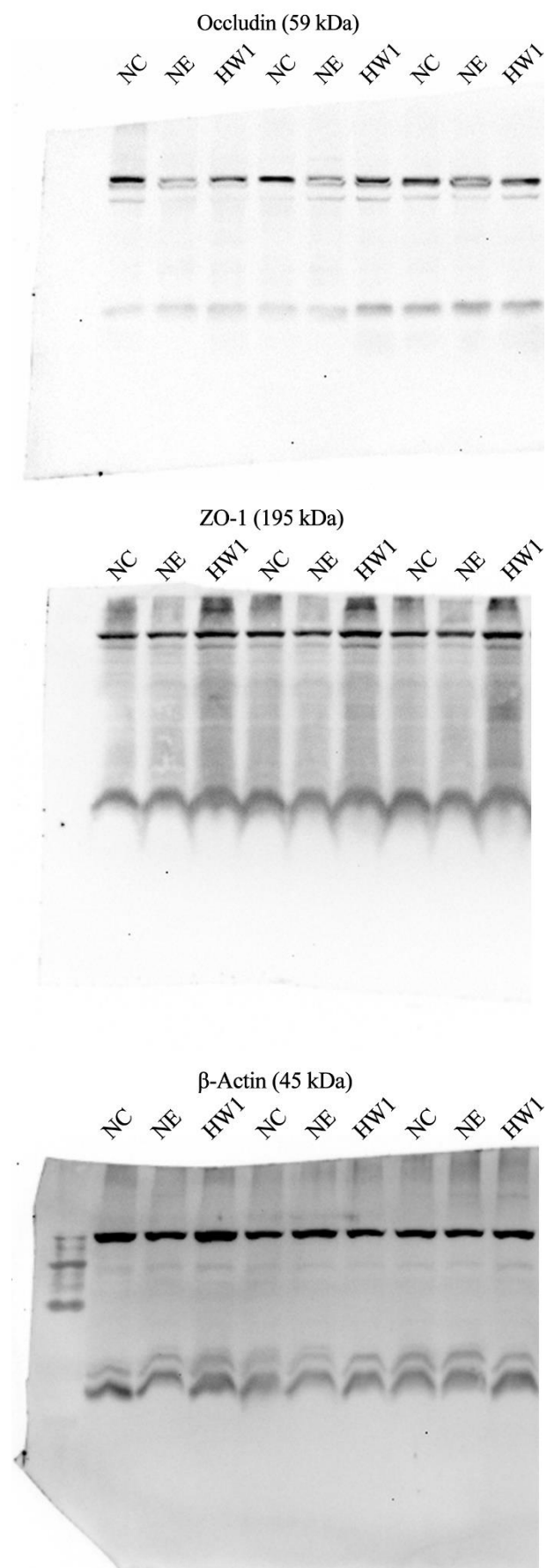

Supplement: Supplementary file 1 [file animals-13-03810-s001.zip › animals-2719445-supplementary.pdf]
